# Will Dentistry Be Absorbed by Medicine?

**Published:** 1895-08

**Authors:** 


### Editorial.

### WILL DENTISTRY BE ABSORBED BY MEDICINE?

THE influences which have been brought to bear upon dentistry to force it to become a specialty of medicine have been powerful and persistent, and in the end will doubtless conquer, and it will cease to be known as a distinct profession.

To the general mind this will not be a matter for regret. Indeed, it will doubtless be welcomed as the legitimate end of all the efforts of the past. While this is in a measure true, it is questionable whether the result will not be attained at the sacrifice of many, if not the majority, of the qualities that have made American dentistry worthy the name it has received.

The status of collegiate instruction in this country in the first period of the organization of dental colleges was necessarily crude and had very little of the character developed at a later stage. The instruction in those branches affiliating with medicine was of the most elementary character, and led to nothing further than a very thin veneer of medical science upon a somewhat disjointed mechanical training.

Further developments came very slowly; indeed, it may be said that for over twenty-five years the colleges, limited in number, were equally limited in resources and gave their students little or nothing worthy the name of medical science. The change that eventually came was in a rapid development, largely induced by organization and a higher appreciation of what a dentist should be to worthily practise.

This development of the scientific side of dental life superinduced a strenuous effort, on the part of many, to seek affiliation with medical men in some of their local organizations, resulting finally in the formation of a section of oral surgery in the National Medical Association and a similar branch in the International Medical Congress. Further than this there has been no advance, and these efforts cannot be regarded as having had any direct influence to bring about closer relations between medicine and dentistry.

While this is true and must ever be true of all forced efforts, it is undoubtedly the fact that the gradual advance of dental education along medical lines has been so rapid and so thorough that the young dentist of to-day who graduates from any one of our leading colleges is capable of standing side by side with his medical *confrère* without any loss of self-respect. This continued and increasing elevation of the standard can have but one result, the destruction of all discordant elements and the breaking down of long-standing prejudices.

What will be the effect of this change? It must be evident that the increased amount of time spent in the acquisition of the foundation sciences—physics, chemistry, anatomy, physiology, histology, pathology, materia medica, and therapeutics—must necessarily reduce the time formerly devoted to the mechanical with its ever-increasing problems in metal and rubber work, crown and bridge insertions, continuous gum and the higher work of orthodontia, or with operative dentistry so important to the best interests of the dentist and his patients.

The necessity for thoroughness in the practical side of a dentist's life seems to require that the dental schools should ever remain as they are distinctly organized for special work. So soon as they become absolutely merged into the medical will the practical part of a dental education suffer degeneration. The absolute absorption of the dental colleges by the medical should never for a moment be tolerated. Separation is the only defence dental education has against the weakness sure to follow any such intermingling.

Many good men in dentistry view this in a different light and hold to the idea that medical education must precede the dental; but experience has amply demonstrated that the adoption of this method of training very rarely results satisfactorily. The long course now required to complete medical studies unfits the student for mechanical details, and at the same time produces a distaste for that kind of work, and skill, except in very rare instances, is never acquired.

There is a growing disposition to place medical men at the head of dental colleges, and while this has not assumed large proportions, it is at present a menace to the well-being of dental schools. While the value and good services of those already connected in this way with dental education is recognized, their presence at the head of dental institutions must be regarded as a serious mistake, and it would seem the duty of the National Association of Dental Faculties to at least recommend to the colleges connected with it to abandon the practice. If this be not done the time is not far distant when dental colleges as distinct organizations will cease to be.

Dental training began by force of medical prejudice in separate dental schools. The utility of this has been amply demonstrated, and there seems no reason now why this course should be changed. Dental colleges are supposed to be paying institutions, and hence the desire to attach these to medical colleges, and while this is well and of mutual advantage, it will require careful guarding lest the great work which has cost so many years of labor be not destroyed in a generation.

